# Molecular Engineering of Polyoxovanadate-Alkoxide Clusters and Microporous Polymer Membranes to Prevent Crossover in Redox-Flow Batteries

**DOI:** 10.1021/acsami.1c23205

**Published:** 2022-02-17

**Authors:** Eric Schreiber, Rachel E. Garwick, Miranda J. Baran, Michael A. Baird, Brett A. Helms, Ellen M. Matson

**Affiliations:** † Department of Chemistry, University of Rochester, Rochester, New York 14627, United States; ‡ Department of Chemistry, University of California, Berkeley, Berkeley, California 94720, United States; § Joint Center for Energy Storage Research, Lawrence Berkeley National Laboratory, Berkeley, California 94720, United States; ∥ The Molecular Foundry, Lawrence Berkeley National Laboratory, Berkeley, California 94720, United States; ⊥ Materials Sciences Division, Lawrence Berkeley National Laboratory, Berkeley, California 94720, United States

**Keywords:** crossover, polyoxometalate, vanadium redox flow battery, polymers of intrinsic microporosity

## Abstract

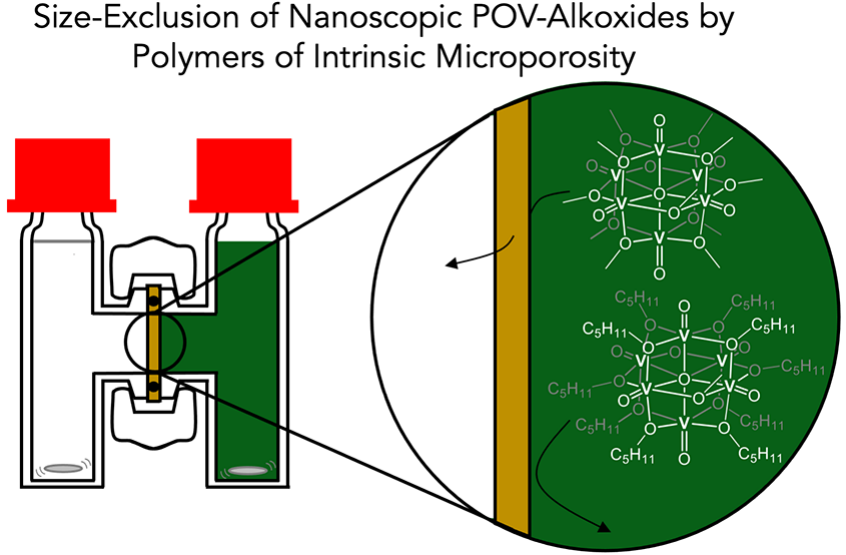

The ongoing development of redox-active charge carriers for nonaqueous redox-flow batteries has led to energy-dense storage concepts and chemistries with high cell voltages. However, rarely are these candidates for flowable energy storage evaluated in tandem with cell separators compatible with organic solvent, limiting progress in the identification of suitable charge carrier–separator pairings. This is important, as the efficiency of a redox-flow battery is dictated by extent of active species crossover through a separator, dividing the two cells, and the contribution of the separator to cell resistance. Here, we report the size-dependent crossover behavior of a series of redox-active vanadium(III) acetoacetonate, and two polyoxovanadate-alkoxide clusters, [V_6_O_7_(OR)_12_] (R = CH_3_, C_5_H_11_) through separators derived from polymers of intrinsic microporosity (PIMs). We find that highly efficacious active-material blocking requires both increasing the size of the vanadium species and restricting pore swelling of the PIMs in nonaqueous electrolyte. Notably, increasing the size of the vanadium species does not significantly affect its redox reversibility, and reducing swelling decreases the conductivity of the separator by only 50%. By pairing polyoxometalate clusters with PIM membranes in nonaqueous redox-flow batteries, more efficient systems may well be within reach.

## Introduction

The growing energy demands of society underscore a critical need for a more resilient electric grid. In this context, advances in grid-scale electrochemical energy storage (EES) are critically important to enable the broad adoption of renewable sources.^1^ Redox-flow batteries (RFBs) are a promising solution for EES. The technology is centered around an electrochemical cell, where redox-active charge carriers are electrochemically cycled, storing electrical energy in the form of disparate redox reactions occurring in the posolyte and negolyte.^2−4^ This introduces a limitation of the RFB schematic: redox-active charge carrier crossover through the separator. This phenomenon lowers the efficiency of the battery during operation due to charge–charge recombination that can sometimes lead to irreversible degradation of the charge carriers.^5^

Nonaqueous redox-flow batteries (NRFBs) are attractive solutions for EES because of their large voltage windows and ability to operate over a wide range of temperatures.^6−9^ However, nonaqueous electrochemical cell separators have lagged behind the development of active species for this technology. Commercially available ion exchange membranes developed for use in aqueous electrochemical energy storage systems (e.g., Nafion, Neosepta, Fumacep, etc.), have been assessed in NRFB schematics; however, low conductivity and poor long-term stability have limited their practical applicability.^5^ Modification to separator materials by pore in-filling with polymers containing zwitterionic functionalities^10^ and subsequent electrical treatment^10,11^ has yielded composite ion exchange membranes which facilitate electrolyte ion conductivity and improve Coulombic efficiencies in organic media, indicating enhanced active species blocking on the part of the membrane. In addition, the development of free-standing, noncommercial ion exchange membranes with charge carrier retention properties have revealed that, by departing from commercial materials, ion exchange membranes with nonaqueous solvent compatibility can be accessed.^12^ On the other hand, porous membranes like Daramic and Celgard provide both conductivity and stability in organic solvents.^5^ Unfortunately, the large pore size of these materials renders them incapable of providing suitable selectivity against charge carrier diffusion, as the size of charge carriers is often significantly smaller than the pore dimension. For these materials, thicker membranes are required to attain sufficient active species blocking. This comes with reduction in electrolyte conductivity through the membrane, resulting in low voltage efficiency for the system.^5^

Several approaches have been investigated to improve the size-selectivity of separators. Strategies typically involve modification to commercially available Celgard; coatings composed of metal organic frameworks,^13^ zeolites,^14^ polysilesquioxanes,^15^ and other fibrous layers^16^ have been explored in this context. Similarly, polymers of intrinsic microporosity (PIMs) have been shown to reduce the diffusivity of charge carriers by up to 5 orders of magnitude relative to that in the electrolyte, whereas salt diffusivity is generally decreased by only 2 orders of magnitude.^17−19^ PIMs have the advantage that they may be processed as defect-free separators with thicknesses of 10–500 μm, or on macroporous substrates as conformal layers generally with thicknesses of 0.5–10 μm. This aids in minimizing nonselective intergrain transport of redox-active charge carriers while also providing a means to minimize the area-specific resistance of the separator, increasing the Coulombic and voltage efficiency in stride. Diversity-oriented approaches to tailoring the pore architectures of PIM separators are also available, granting access to high-transference numbers optionally for cation or anion transport, depending on the types of ion cages introduced.^20^ PIM-1 and its cross-linked form (XPIM-1) are the most studied microporous polymer separators to date; both size- and chemistry-selective mechanisms have been observed in reducing crossover of the redox-active charge carriers. With respect to the former, the small pore size (6–11 Å)^21^ and nonaqueous solvent compatibility of this separator hinder crossover of organic redox-active molecules, oligomers, polymers, and colloids above the exclusion limit of the separator, even after hundreds of hours.^17−19^ However, to date, few studies on the inhibition of separator crossover by size-exclusion membranes for vanadium NRFBs have been presented,^16,22−24^ and none have been conducted using PIM-derived separators.

**Figure 1:**
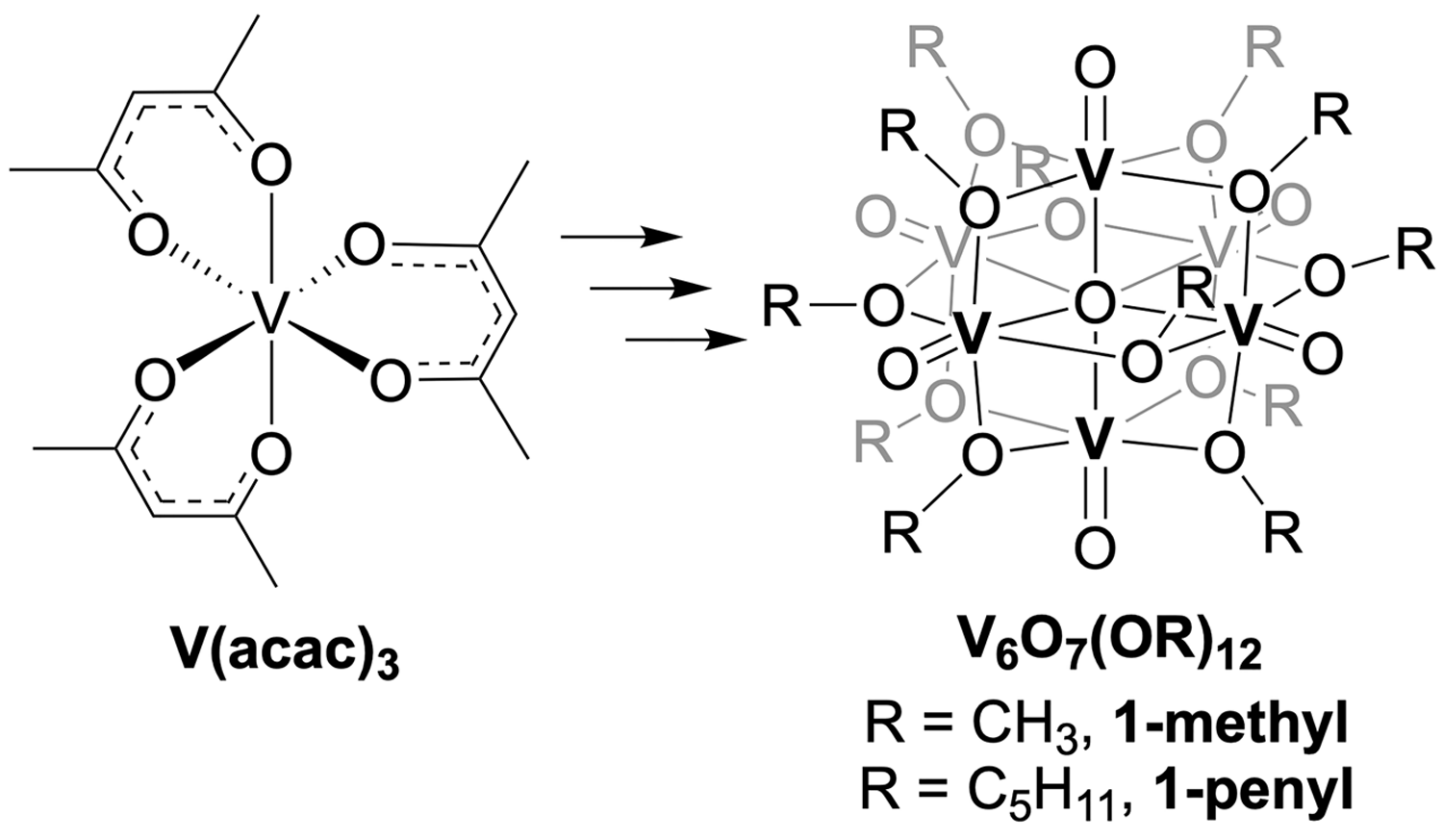
Vanadium-based, redox-active charge carriers studied in this work for their resistance to crossover in separators coated with polymers of intrinsic microporosity for nonaqueous redox-flow batteries.

Herein, we report a kinetic study on the crossover of a series of inorganic charge carriers through PIM-1-coated Celgard 2400 separators to assess the effect that molecular size, shape, and nuclearity has on the crossover of metal-derived active species (Figure 1). The canonical metal-containing charge carrier in NRFB research is vanadium(III) acetylacetonate (**V(acac)**_**3**_); this molecule and its derivatives have been applied in a variety of NRFB studies, with a principal factor influencing the loss of efficiency cited as self-discharge due to active species crossover.^16,22,25−29^ Drawing inspiration from the design strategy of generating organic oligomers, we opted also to explore membrane crossover of multinuclear, organo-functionalized vanadium oxide assemblies, namely polyoxovanadate-alkoxides (POV-alkoxides; Figure 1). These clusters are comprised of six vanadyl ions bridged by alkoxide ligands, which stabilize reduced forms of the cluster and afford the metal oxide core solubility in organic solvent. In recent work, we have expanded the library of known derivatives of this complex to include variants with longer, aliphatic alkoxide ligands, demonstrating the tunability of size of the metal oxide assembly. Notably, deviations in the molecular dimensions and mass do not significantly influence bulk electrolyte diffusivity or heterogeneous charge transfer rate with the current collector, identifying this family of complexes as prime candidates for studying membrane crossover characteristics for NRFB active species as a function of the chain-length of surface alkoxide ligands. We find that by controlling the cluster size, surface chemistry, and membrane swelling, highly efficacious active-species blocking becomes feasible, whereas conventional vanadium compounds rapidly crossover. Our results further the collective understanding of how to synergistically meld the molecular and device component design to enhance battery performance.

## Results and Discussion

Interested in the dynamics of inorganic charge carrier crossover in size-exclusion membranes with relevance for NRFB schematics, we performed experiments designed to determine the rates of through-separator diffusion for a series of redox-active vanadium complexes, including **V(acac)**_**3**_ and two POV-alkoxide clusters with differing surface ligands. The Lindqvist-type POV-alkoxide clusters, V_6_O_7_(OCH_3_)_12_ (**1-methyl**), and V_6_O_7_(OC_5_H_11_)_12_ (**1-pentyl**) have been studied by our lab as candidates for NRFBs, with **1-pentyl** identified as the complex with the longest hydrocarbon-capping ligands that still allow for acceptable electrochemical performance in NRFB cycling experiments.^30,31^

**Figure 2:**
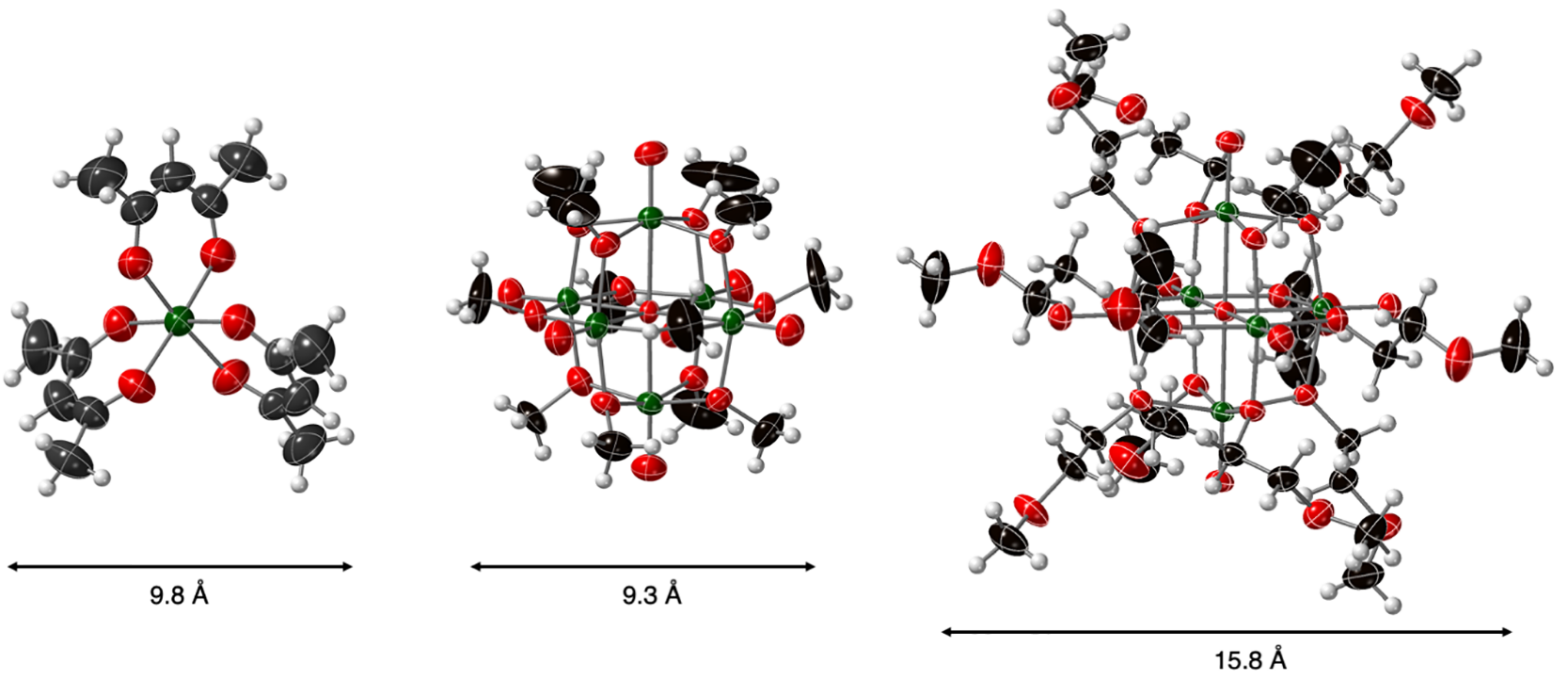
Molecular structures of **V(acac)**_**3**_, **1-methyl**, and V_6_O_7_(OC_2_H_5_OCH_3_)_12_ shown with 30% probability ellipsoids. Molecular diameters were estimated by taking an average of the distances between the furthest opposing hydrogen atoms on the exterior of each complex.

With relevance to the membranes selected for investigation in this work, molecular size has been established to be a key design criterion for minimization of crossover in NRFBs. The three complexes investigated here possess varying molecular structures and sizes that we anticipated would influence crossover through size-selective membranes (Figure 2). **V(acac)**_**3**_ has three bulky acetoacetonate ligands, resulting in a molecular diameter of 9.8 Å; the large size of this molecule has been hypothesized to partially mitigate membrane crossover in previous studies.^32^ The hexavanadate assembly featuring methoxide ligands at its surface, by comparison, has an approximate diameter of 9.3 Å in the solid phase, according to single crystal X-ray diffraction (SCXRD).^33^ The larger vanadium oxide assembly, **1-pentyl**, is a viscous oil at room temperature, and therefore cannot be directly studied by SCXRD.^31^ However, a cluster derived from 2-methoxyethanol capping ligands, V_6_O_7_(OC_2_H_5_OCH_3_)_12_, has been crystallized and possesses a diameter of 15.8 Å.^34^ Being that this compound comprises a 4-atom chain as opposed to the five carbon-chain of pentanol, we anticipate that **1-pentyl** will have a slightly larger molecular diameter (>16 Å).

To assess the size-dependence on through-separator diffusion of **V(acac)**_**3**_, **1-methyl**, and **1-pentyl**, we constructed H-cells that contained solutions of electrolyte (0.1 M [^n^Bu_4_N][PF_6_] in MeCN) with active species and electrolyte at a specified concentration (see the Supporting Information). Additional [^n^Bu_4_N][PF_6_] was added to the permeate side of the cell to maintain an osmotic balance during the experiment. The two half-cells were isolated by one of three separators: either bare Celgard 2400, native PIM-1-coated Celgard 2400 (**PIM-1**), or PIM-1-coated Celgard that has been cross-linked with 2,6-bis(4-azidobenzylidene)cyclohexanone (**XPIM-1**). The extrinsic rate of crossover was determined by the slope of concentration vs time, where concentration was quantified electrochemically using cyclic voltammetry (CV) of the permeate and a calibration curve in the relevant concentration regime for the redox-active charge carrier in electrolyte (Figure S1). Taking into account the cell architecture, we determined the intrinsic (i.e., effective) diffusivity (*D*_eff_) of the redox-active charge carrier through **PIM-1** and **X-PIM-1**, which we compare to the diffusivity of the species solubilized in electrolyte (*D*_sol_) as the figure of merit for the blocking ability of the separator.

### Diffusion of Charge Carriers through Celgard 2400

As the foundation of the study, we determined the diffusivity of all three active species through a nonselective mesoporous separator, Celgard 2400. This separator is made from polypropylene and features 430 Å pores. Celgard 2400 is compatible with a diverse range of nonaqueous electrolytes.^35−37^ We also used Celgard 2400 as the support for the active-material blocking PIM overlayers: **PIM-1** and **XPIM-1**.

**Figure 3:**
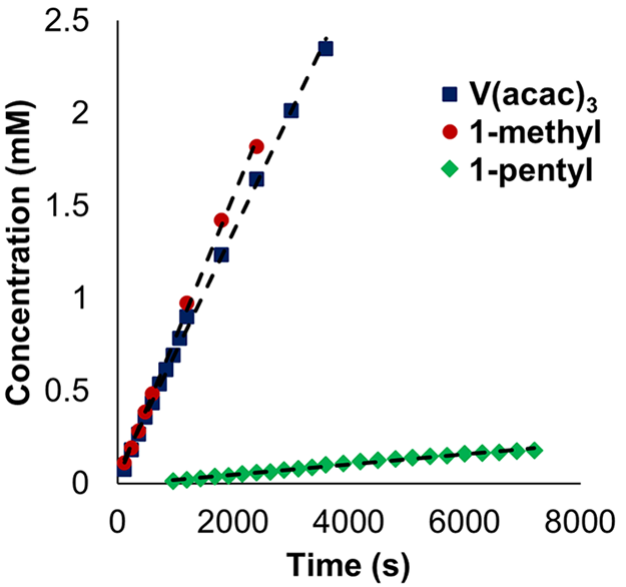
Concentration of permeate species ****V(acac)**_**3**_** (blue triangles), **1-methyl** (red circles), and **1-pentyl** (green diamonds) as a function of stir time in crossover experiments with bare Celgard 2400 separators. Initial concentration of retentate species: **V(acac)**_**3**_ (10 mM), **1-methyl** (10 mM), and **1-pentyl** (4 mM).

As all molecules selected here for analysis feature smaller diameters than the pores of Celgard 2400, we anticipated that diffusion through the bare membrane would be rapid. As expected, detectable amounts of crossover of both **V(acac)**_**3**_ and **1-methyl** occurred at early time points (∼2 min; Figure 3). For **1-pentyl**, observable crossover was not detected until 15 min of stirring time; the delayed crossover indicates that the increased size of the POV-alkoxide as a result of incorporation of bulky pentanol-derived surface ligands decreases the diffusivity of the cluster across the unmodified Celgard 2400 (Figure 3).

To compare the through-separator diffusion to the electrolyte diffusivity of the three compounds, we invoke eq 1, which determines the effective diffusion coefficient, *D*_eff_, for a complex through a separator material in cm^2^ s^–1^:^17^
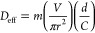
1Where *m* is the slope of the line relating crossover experiment time and active species concentration on the electrolyte-only side (M s^–1^), *V* is the volume of solutions in each half cell (mL), *r* is the aperture radius of the exposed membrane (cm), *d* is the thickness of the membrane (cm), and *C* is the initial active species concentration (M) in the analyte half-cell. Application of eq 1 to data collected for crossover of all three molecules with the Celgard 2400 membrane reveals the *D*_eff_ values listed in Table 1 (in addition, see the Supporting Information).

**Table 1:** Diffusivity of Inorganic Charge Carriers through the Electrochemical Medium and through H-Cell Separators Constructed from Bare Celgard 2400, PIM-1-Coated Celgard, and Cross-Linked PIM-1-Coated Celgard

		Celgard 2400		PIM-1		XPIM-1	
compd	*D*_sol_ × 10^6^^a^ (cm^2^ s^–1^)	*D*_eff_ × 10^6^^b^ (cm^**2**^ s^–1^)	*D*_sol_/*D*_eff_	*D*_eff_ × 10^8^^c^ (cm^2^ s^–1^)	*D*_sol_/*D*_eff_	*D*_eff_ × 10^9^^c^ (cm^2^ s^–1^)	*D*_sol_/*D*_eff_
**V(acac)**_**3**_	6.2^26^	0.975	6.36	1.54	402	8.48	440
**1-methyl**	1.4^27^	1.18	1.19	0.487	287	3.13	436
**1-pentyl**	1.76^28^	0.105	16.8	0.0544	3,237	0.0633	17,150

a *D*_sol_ is the diffusivity of the inorganic complex within the MeCN electrolyte.

b *D*_eff_ for Celgard is the diffusivity of a compound through a bare Celgard 2400 sheet.

c *D*_eff_ for **PIM-1** and **XPIM-1** are the diffusivities of a complex through the polymer coating in isolation after accounting for diffusion through Celgard.

These results show a modest size-dependence in active species diffusivity through the microporous separator, where species with the large molecular diameters (e.g., **1-pentyl**) diffuse with sluggish kinetics while smaller compounds (e.g., **V(acac)**_**3**_, **1-methyl**) exhibit rapid crossover. Comparison of these values with the diffusion coefficients for these molecules through the relevant electrolyte (*D*_sol_; values determined using the Randles–Sevcik equation^29−31^) further demonstrates this point. This ratio serves to highlight the order by which active species diffusion from one-half cell to the other is slowed by the separator. In the absence of a separator material, diffusion would be described by *D*_sol_, or the electrolyte diffusivity; this is the fastest rate at which the molecules diffuse through solution. The introduction of a physical barrier should result in reduced diffusion rates, producing a *D*_sol_/*D*_eff_ ratio greater than 1. Consideration of these ratios allows us to gauge diffusion of each molecule through the membrane versus their fastest diffusion rates (*D*_sol_). Values for *D*_sol_/*D*_eff_ were 1.19, 6.36, and 16.8 for **1-methyl**, **V(acac)**_**3**_, and **1-pentyl**, respectively (Table 1).

Although the general observation of the crossover rates for these complexes follows the expected trend for molecular diameter, the comparable crossover rates between **1-methyl** and **V(acac)**_**3**_ are in contrast a previous report from our research group. In that work, it was revealed that the methoxide-bridged POV-alkoxide diffuses more slowly than **V(acac)**_**3**_ through an AMI-7001 separator.^30^ Notably, this commercially available separator is an anion exchange membrane, and therefore operates on alternative principles to that of Celgard. As such, we credit the inverted trend in crossover rate observed for **V(acac)**_**3**_ and **1-methyl** to differences in separator chemistries.

We credit the poor blocking performance observed with Celgard 2400 to its large pore size, which does not provide sufficient size-selectivity for the complexes of interest (all charge carriers fall below 4% of the pore diameter). This translates to diffusion through the separator at comparable rates to mass transport through electrolyte. Previous work on active-species crossover through Celgard 2400 revealed that only when the diameter of the active species reached 14 nm, or 33% of the Celgard pore size, did size-exclusion prove effective.^36^ The seemingly significant improvement in blocking when comparing *D*_eff_ values determined for **1-methyl** and **1-pentyl** is therefore surprising considering the molecular diameters of these clusters. Importantly, initial analyte concentration is accounted for in determination of *D*_eff_, meaning this is an intrinsic value that is unique to each electrolyte, separator, and analyte combination (see the Supporting Information for details). Thus, the differences in *D*_eff_ are not a consequence of initial retentate concentration. In addition, the order of magnitude slower rate of diffusion for **1-pentyl** versus **1-methyl** still falls in the 1 × 10^7^ cm^2^ s^–1^ regime, which in comparison to other examples of active species crossover rates in nonaqueous systems, represents rapid diffusion of the compound through the porous membrane.^17−19^

### Crossover Performance across **PIM-1**-Coated Separators

**Figure 4:**
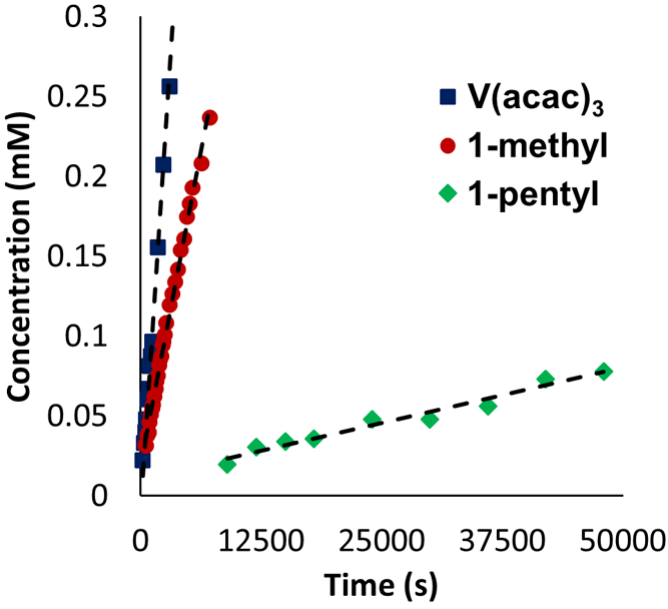
Concentration of permeate species **1-methyl** (red circles), **V(acac)**_**3**_ (blue triangles), and **1-pentyl** (green diamonds) as a function of stir time in crossover experiments with **PIM-1**-coated separators. Initial concentration of retentate species: **V(acac)**_**3**_ (10 mM), **1-methyl** (10 mM), and **1-pentyl** (4 mM).

To improve charge species retention, we turned to the investigation of **PIM-1** membranes, which feature pore sizes nominally in the range of 6–11 Å.^21^ Similar crossover experiments were performed to those with Celgard 2400 with all three inorganic complexes, where diffusion was hindered by comparison to the control (i.e., the nonblocking Celgard separator; Table 1). **V(acac)**_**3**_ was observed to penetrate the membrane within 5 min, indicating little change in the extrinsic crossover rate. *D*_eff_ for **V(acac)**_**3**_ through the layered hybrid separator was only slower by a factor of 6.65 compared to the control (Figure 4, Table 1). However, when considering diffusion through PIM-1 in isolation, we note that the intrinsic diffusivity of **V(acac)**_**3**_ is hindered by a further order of magnitude, with a relative *D*_eff_ 63 times lower than Celgard 2400, producing a *D*_sol_/*D*_eff_ of 402 for the PIM-1 coating (Table 1).

Similar crossover behavior was observed for the methoxide-bridged **1-methyl**, wherein the diffusivity of this complex through **PIM-1** was slowed by a factor of 23 for the composite material, resulting in a *D*_sol_/*D*_eff_ of 287 for this molecule through the polymer film (Figure 4, Table 1).

Where the native polymer-coated separator resulted in modest blocking for complexes **V(acac)**_**3**_ and **1-methyl**, migration of **1-pentyl** was found to be substantially inhibited. Only after 2.5 h did the concentration of **1-pentyl** reach the limit of detection of our experiment (Figure 4). This represents a significant improvement over unmodified Celgard 2400, which reached the limit of detection by 16 min. In addition, diffusivity through the layered separator revealed a 23-fold decrease in crossover rate, and a *D*_eff_ for the polymer region of the membrane 3240 times lower than electrolyte diffusion, representing the highest performance for the complexes in this study on the native PIM-1-coated Celgard 2400 separators.

These observations indicate that active-species crossover of inorganic complexes is restricted by the polymer coating. For both **V(acac)**_**3**_ and **1-methyl**, the introduction of the coating imparted only slight size selectivity to the separator. This is a consequence of the comparable size of both molecules to the pores of the **PIM-1** membrane, resulting in facile diffusion of active species molecules through the separator. With its larger molecular diameter, **1-pentyl** was blocked by the **PIM-1** membrane much more effectively.

Crossover of **1-pentyl** through **PIM-1** suggests that the separator exclusion limit in the H-cell is larger than that suggested by the pore size distribution measured for **PIM-1** in the dry state. This is due to the swelling of PIM-1 in nonaqueous electrolyte. To reduce swelling in PIM-1, we introduced 2,6-bis(4-azidobenzylidene)cyclohexanone as a cross-linker. This was accomplished by dissolving the cross-linker in the PIM-1 ink prior to casting on the support and invoking a UV-light initiated nitrene insertion into C–H bonds of the native polymer at 365 nm (Figures S1 and S2). This strategy is an alternative to thermal methods previously reported.^17^**XPIM-1** features increased rigidity, further restricting pore size deviations upon exposure to solvent, which has been shown to enhance separator performance.^17,19^ Thus, applying **XPIM-1** in crossover experiments should result in further reductions in diffusive permeability for the selected charge carriers through the separator.

**Figure 5:**
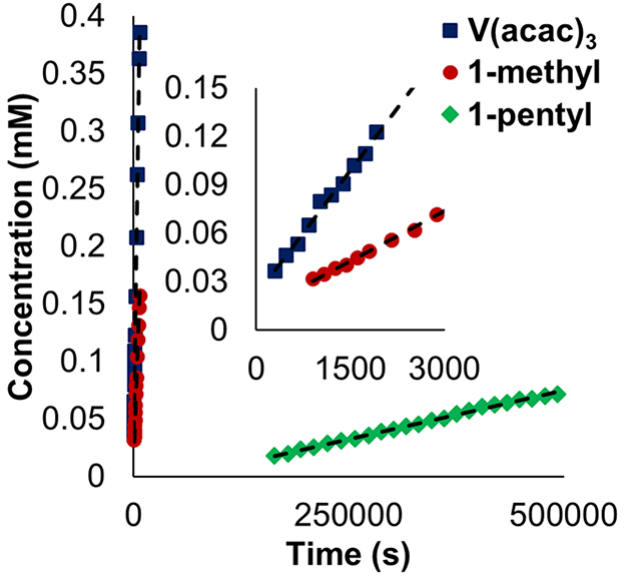
Concentration of permeate species **1-methyl** (red circles), **V(acac)**_**3**_ (blue triangles), and **1-pentyl** (green diamonds) as a function of stir time in crossover experiments with **XPIM-1** coated separators. Initial concentration of retentate species: **V(acac)**_**3**_ (10 mM), **1-methyl** (10 mM), and **1-pentyl** (4 mM).

In crossover experiments for both **V(acac)**_**3**_ and **1-methyl** with **XPIM-1** separators, only a slight decrease in diffusive permeability was observed (Figure 5, Table 1). It is worth noting that in both cases, the complexes are of comparable size to the micropores of the polymer coating, meaning that the reduced, but nonzero expansion in pore diameter allowed by the cross-linking process, still allows for passage of these complexes at high rates. This is a surprising result considering molecules of similar diameter have been previously observed to be blocked more effectively by cross-linked PIM-1 membranes (*D*_sol_/*D*_eff_ ∼ 1 × 10^4^).^17,19^ We attribute the observed crossover behavior to the high molecular symmetry of the inorganic complexes in this study. The compact and rigid shape of these complexes facilitates partitioning, despite restrictions imposed by smaller pore apertures, whereas similarly sized organics with linear and branched architectures studied with cross-linked PIM-1 separators in the past adopt a range of conformations, allowing for improved blocking.^17−19^

On the other hand, analysis of the crossover performance of **1-pentyl** with **XPIM-1** reveals a substantial improvement in retention of the electroactive species (Figure 5, Table 1). First, monitoring the cyclic voltammogram of the permeate half-cell revealed that prior to 45 h of stirring, diffused analyte remained below detectable levels, after which point the concentration increased linearly with time (Figure 5). In addition, after 136 h, only 1.8% of the cluster had permeated the membrane, indicating good performance and reflecting a 6.4-fold improvement in *D*_eff_ for the composite **XPIM-1** system over **PIM-1** (Table 1). When considering the thin, 3.24-μm layer of cross-linked polymer, this performance yields a *D*_sol_/*D*_eff_ of 17 150, which coupled with the time required to reach the limit of detection reveals a high-performing charge carrier–separator pairing.

The excellent blocking ability of the **XPIM-1** membrane necessitates assessment of its ionic conductivity. This is to determine if the membrane is selective for active species blocking, while electrolyte is able to freely flow through the separator. Analysis of the electrochemical impedance spectra for the tetrabutylammonium hexafluorophosphate solution in MeCN, with Celgard 2400 and **XPIM-1** separators, revealed a 2-fold decrease in ionic conductivity for the coated separator, indicating that adequate ionic conductivity was retained upon coating with the microporous polymer (Figure S8, Table S5).

**Figure 6:**
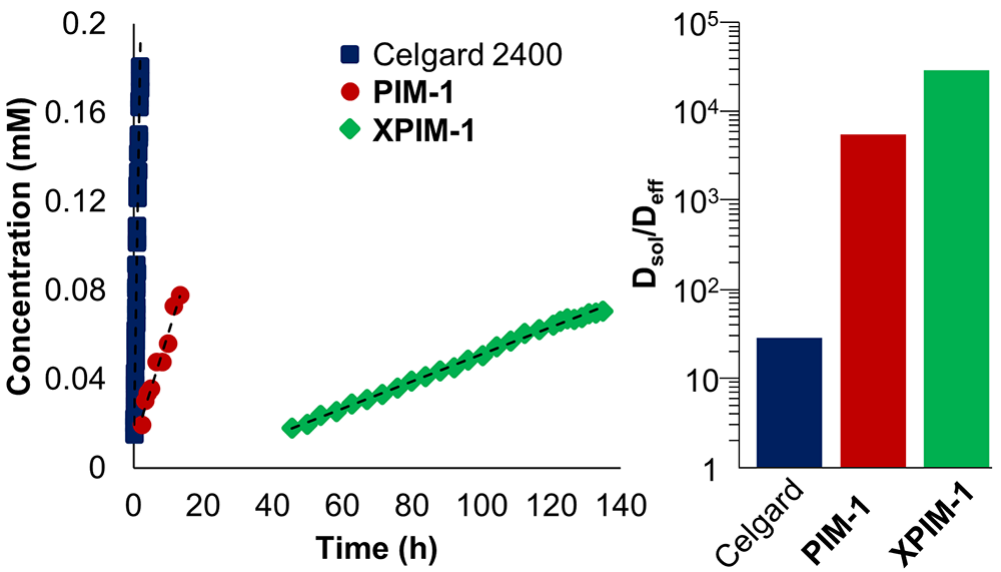
(Left) Crossover data for **1-pentyl** through Celgard 2400, **PIM-1**, and **XPIM-**1, and (right) membrane blocking ability of each membrane for this compound. *D*_sol_/*D*_eff_ describes the degree by which through-membrane diffusion is slower than through-electrolyte mass transport. All initial retentate concentrations for **1-pentyl** are 4 mM.

Where the observed reduction in crossover kinetics for the **1-pentyl** and **XPIM-1** system reveals synergistically optimized system for maintaining isolated RFB compartments (Figure 6), the observed *D*_sol_/*D*_eff_ is slightly lower than previously studied organic charge carriers of comparable molecular diameters.^17^ This is likely due to the flexibility of the surface ligands on **1-pentyl**. Previous work on organic charge carriers has studied oligomeric viologen charge carriers that contain rigid aromatic structures and short linker groups, imparting rigidity to the overall structure. Indeed, considering the trimeric oligomer (diameter: 16.8 Å), which features a symmetrical, rigid structure, crossover experiments with cross-linked PIM-1 separators revealed that the permeated compounds never reached concentrations above the limit of detection after extended timeframes, yielding an estimated *D*_sol_/*D*_eff_ of 84 500.^17^ The flexibility of the aliphatic organic moieties that flank the surface of **1-pentyl**, in contrast, enable this cluster to adopt smaller conformations than that predicted for the cluster.

## Conclusion

Vanadium remains the most widely used active material for redox-flow batteries. Commercial redox-flow batteries implementing vanadium use aqueous electrolytes and are limited by that choice with respect to cell voltage and energy density. Alternative designs implementing nonaqueous flowable electrolytes have been widely studied, where it was found that conventional separators and ion exchange membranes failed to prevent crossover, lowering the energy and voltage efficiency of those systems. To advance the state-of-the-art, a more detailed molecular understanding of the crossover behavior in nonaqueous redox-flow batteries is warranted. To that end, we have systematically explored the relationship between the size of the charge carrier—vis-à-vis molecular complexes of vanadium as well as oxo clusters thereof varying in size up to 1.6 nm—alongside the pore size of the separator—vis-à-vis polymers of intrinsic microporosity that feature subnanometer pores. Through detailed transport and electrochemical studies, we have demonstrated that it is possible through molecular engineering to increase the size of the redox-active vanadium species to be at or above the exclusion limit for PIM separator without significantly affect its redox reversibility in nonaqueous electrolyte. Our success hinged on restricting the pore dimensions of the PIM separator from swelling excessively, which we accomplished by using a cross-linker. Chemical cross-linking of the PIM separator only increased its area-specific resistance by a factor of 2, maintaining a low value that bodes well for future applications in flow cells. We further see exciting opportunities to study state-of-charge-dependent crossover of the clusters through PIM separators, because of their availability in different states of charge.^30,31^ Nanoscale engineering continues to play a major role in redox-flow battery development as we develop practical systems that can scale to meet the growing need for energy storage solutions for the grid, from multihour to long duration.

## Experimental Section

### General Considerations

All manipulations, unless otherwise noted, were carried out in the absence of water and oxygen in an inert atmosphere glovebox under a nitrogen or argon atmosphere (UniLab MBraun, N_2_ or Ar). Glassware was oven-dried for a minimum of 4 h and cooled in an evacuated antechamber prior to use. Three angstrom molecular sieves (Fisher Scientific) were dried in a Schlenk flask for 48 h at 125 °C under vacuum prior to use. Acetonitrile was dried and deoxygenated on a Glass Contour System (Pure Process Technology, LLC) and stored over activated 3 Å molecular sieves or purchased from Sigma-Aldrich as anhydrous and stored over activated 3 Å molecular sieves. Tetrabutylammonium hexafluorophosphate (TBAPF_6_) was purchased from Sigma-Aldrich and recrystallized three times from hot ethanol and stored under a dynamic vacuum for at least 24 h prior to use. Vanadium *tris*(acetylactonate) (**V(acac)**_**3**_) was purchased from Sigma-Aldrich and used as received. Complexes [V_6_O_7_(OCH_3_)_12_] (**1-methyl**)^33^ and [V_6_O_7_(OC_5_H_11_)_12_] (**1-pentyl**)^31^ were synthesized according to reported procedures. PIM-1 was synthesized as described elsewhere.^38−40^

Two-part H-cells with 1.6 cm diameter apertures were purchased from Adams and Chittenden Scientific Glass. Glassy carbon electrodes and nonaqueous Ag/Ag^+^ reference cell electrodes were purchased from BAS Inc. Reference electrodes were filled with 0.01 M silver nitrate (AgNO_3_) in 0.1 M TBAPF_6_. Electrochemical measurements using Celgard and **PIM-1** separators were performed using a Bio-Logic SP-300 potentiostat. Electrochemical measurements using **XPIM-1** were performed using a Bio-Logic VMP3 potentiostat. Cyclic voltammograms were measured at 100 mV s^–1^ with IR compensation accounted for by measuring the uncompensated resistance with a 100 kHz impedance measurement and correcting for 85% of the expected drop.

### Preparation of Electrolyte Solutions

Distinct electrolyte solutions were made for the two compartments of the cell. One solution (the retentate) was made using 0.1 M TBAPF_6_ and 10 mM of selected compound (due to solubility issues the solution containing V_6_O_7_(OC_5_H_11_)_12_ contained 4 mM of compound) in 12 mL of acetonitrile. The second solution (the permeate) was made using 0.105 or 0.102 M TBAPF_6_ in 12 mL of acetonitrile to account to provide initial osmotic balance between the two compartments.

### Preparation of Coated Separators

Inks of PIM-1 were prepared in chloroform (175 mg mL^–1^) were stirred overnight. The ink was blade coated onto one side of sheets of bare Celgard 2400 and dried in vacuo overnight. Membranes were cut using a razor blade into the shape of the H-cell separator compartment, and brought into the glovebox. All separators (including bare Celgard 2400) were soaked in electrolyte solution overnight prior to use. For cross-linking, 10 mol % (with respect to monomer) of 2,6-bis(4-azidobenzylidene)cyclohexanone was added to the ink prior to casting. Once dried, sheets of coated Celgard 2400 were cross-linked by irradiation with UV light (λ = 365 nm) for 30 min, at which time the IR feature corresponding to the azide group in the cross-linker had been completely diminished (Figures S1 and S2). Coating quality was confirmed by analysis on a Gurley Precision Instruments 4150N high pressure densometer (Table S1).

### Scanning Electron Microscopy Imaging of PIM-Coated Celgard 2400

Imaging was performed using a Zeiss Gemini Ultra-55 analytical field emission scanning electron microscope with a 5 kV accelerating voltage and using the secondary detector mode (Figure S3 and Table S1).

### Procedure for Crossover Experiments Using Celgard and PIM-1 Membranes

Calibration curves were determined to relate concentration and current density for each compound using the reversible oxidation event of V(acac)_3_ (Figure S4a), and the first reducing event for the POV-alkoxide clusters (Figure S4b, c). Before each experiment, a CV was taken of both electrolyte solutions ensuring purity of the electrolyte using a 3 mm glassy carbon electrode. A standard H-cell with an aperture diameter of 1.6 cm was fitted with the membrane of choice (standard Celgard, PIM-1, or cross-linked PIM-1) and sealed with a chemically resistant O-ring. The cell was then charged with 2 stir bars, one in each compartment. Each compartment of the cell was filled simultaneously, one side containing permeate and the other side containing retentate. Data were collected by allowing both solutions in the cell to stir for 2–20 min. After the allotted time period, each chamber was simultaneously emptied, and a CV was taken of the permeate. The solutions were simultaneously replaced into each chamber and the process was repeated. CV data collected for these separators are reported in Figures S5a, b, S6a, b, and S7a, b.

### Procedure for Crossover Experiments Using Celgard and PIM-1 Membranes

Using a similar H-cell setup and initial experimental setup to the above crossover experiments, these crossover tests were performed for all three inorganic charge carriers using a potentiostat-controlled stirring apparatus. The solutions were stirred 2–60 min at a time, after which CV were acquired with a 1 mm glassy carbon electrode and the stirring was repeated. The same calibration curves as used in the hand-run crossover studies were used to determine the concentration of carriers in the permeate. CV data collected in these experiments are reported in Figures S5c, S6c, and S7c.

### Membrane Ionic Conductivity Measurements

Soaked membranes were sandwiched between two 1.2 cm stainless steel electrodes in a Swagelok cell. Potentioelectrochemical impedance spectroscopy (PEIS) was used with 10 mV AC bias scanning from 200 kHz to 100 mHz. The high-frequency *x*-axis intercept is taken to be the resistance of the membrane. The membrane conductivity was then calculated by accounting for the cell geometry (Figures S8 and S9 and Table S5).

### Electrolyte Ionic Conductivity Measurements

Solutions of 0.1 M [^n^Bu_4_N]PF_6_, as well as this electrolyte with 10 mM 1-methyl and 4 mM 1-pentyl in MeCN were loaded into an electrochemical cell. A Pt mesh working electrode, Pt wire counter electrode, and Ag wire reference electrode were used for the measurement, separated by 2 mm. Potentioelectrochemical impedance spectroscopy (PEIS) was performed at a working electrode voltage of 0 V versus the open circuit potential, and the frequency was scanned from 200 kHz to 100 mHz with a sinus amplitude of 10 mV. The high-frequency *x*-axis intercept is taken to be the resistance of the electrolyte solution (Figure S10).

## Supporting Information Available

FTIR measurements of UV cross-linking procedure for PIM-1-coated Celgard 2400, calibration curves for crossover experiments, plots of raw CV data from crossover experiments, kinetic details for membrane diffusivity, EIS plots with fits, and conductivity measurements (PDF)
