# Supplementary material for: Molecular Engineering of Polyoxovanadate-Alkoxide Clusters and Microporous Polymer Membranes to Prevent Crossover in Redox-Flow Batteries
Source: ACS Appl Mater Interfaces. 2022 Feb 17;14(20):22965–72. doi: 10.1021/acsami.1c23205 (PMC9136837; doi:10.1021/acsami.1c23205)
Supplement: Supplementary file 1 — am1c23205_si_001.pdf [file am1c23205_si_001.pdf]

Supporting Information

Molecular Engineering of Polyoxovanadate-Alkoxide Clusters and Microporous Polymer Membranes to Prevent Crossover in Redox-Flow Batteries

Eric Schreiber[a], Rachel E. Garwick, Miranda Baran[b,c], Michael A. Baird[b], Brett A. Helms*[c,d,e], and Ellen M. Matson*[a]

[a] Department of Chemistry, University of Rochester, Rochester NY 14627

[b] Department of Chemistry, University of California, Berkeley, Berkeley, CA 94720

[c] Joint Center for Energy Storage Research, Lawrence Berkeley National Laboratory, Berkeley, CA 94720

[d] The Molecular Foundry, Lawrence Berkeley National Laboratory, Berkeley, CA 94720

[e] Materials Sciences Division, Lawrence Berkeley National Laboratory, Berkeley, CA 94720

Corresponding Authors:

Email:

matson@chem.rochester.edu *(E. M. Matson)*

bahelms@lbl.gov *(B. A. Helms)*

Supporting Information Table of contents.

Figure S1. Schematic demonstrating the UV irradiation process invoked for the crosslinking of **PIM-1** coated separators with 2,6-bis(4-azidobenzylidene)cyclohexanone, and the proposed crosslinking mechanism……………………………………………………………………………………….S3

Figure S2. (a) FTIR spectrum of PIM-1-coated Celgard 2400 with 10 mol% 2,6-bis(4-azidobenzylidene)cyclohexanone before (grey) and after (red) exposure to 365 nm light for 30 min. (b) Expanded region of the spectrum to highlight consumption of the azide upon UV light exposure……………...…...……………………………………………………………...……S3

Figure S3. SEM images of polymer-coated separators used in crossover studies with a) **V(acac)$_3$**, b) **1-methyl**, and c) **1-pentyl**. Thicknesses are tabulated in Table S3………………………………...S4

Table S1. SEM measurements and Gurley numbers of **XPIM-1** coated separators used in this study...S4

Figure S4. Calibration curves relating active species concentration and current density for the relevant redox events used in this study for a) **V(acac)$_3$**, b) **1-methyl**, and c) **1-pentyl**…...…...………………..S5

Figure S5. CV data collected in crossover experiments of **V(acac)$_3$** with a) bare Celgard 2400, b) **PIM-1**, and c) **XPIM-1** separators.………………………………………………………………......…S6

Figure S6. CV data collected in crossover experiments of **1-methyl** with a) bare Celgard 2400, b) **PIM-1**, and c) **XPIM-1** separators …………………………………………………………….…….S7

Figure S7. CV data collected in crossover experiments of **1-pentyl** with a) bare Celgard 2400, b) **PIM-1**, and c) **XPIM-1** separators …………………………………………………………….…….S8

Table S2. Crossover parameters for active species on Celgard 2400……………………..………...S9

Table S3. Crossover parameters for active species on **PIM-1**………………………………………...S9

Table S4. Crossover parameters for active species on **XPIM-1**……………………….........……...S9

Figure S8. EIS Spectra of electrolyte-soaked Celgard 2400 (black circles) and **XPIM-1** (red circles) membranes…………………………………...………………………….……………….....…...S10

Figure S9. Equivalent circuit used for EIS fitting of spectra in Figure S8…………………………...S10

Table S5. EIS parameters for conductivity measurements on Celgard 2400 (black circles) and **XPIM-1** (red circles) membranes…….…………………….……………………………………………..S10

Figure S10. a) EIS spectra of electrolyte solutions of 0.1 M [^{n}Bu$_4$N]PF$_6$, as well as this electrolyte with 10 mM 1-methyl and 4 mM 1-pentyl, with their respective solution resistance (R$_s$) values. b) Equivalent circuit used to fit the EIS spectral data………………………………………………………..S11

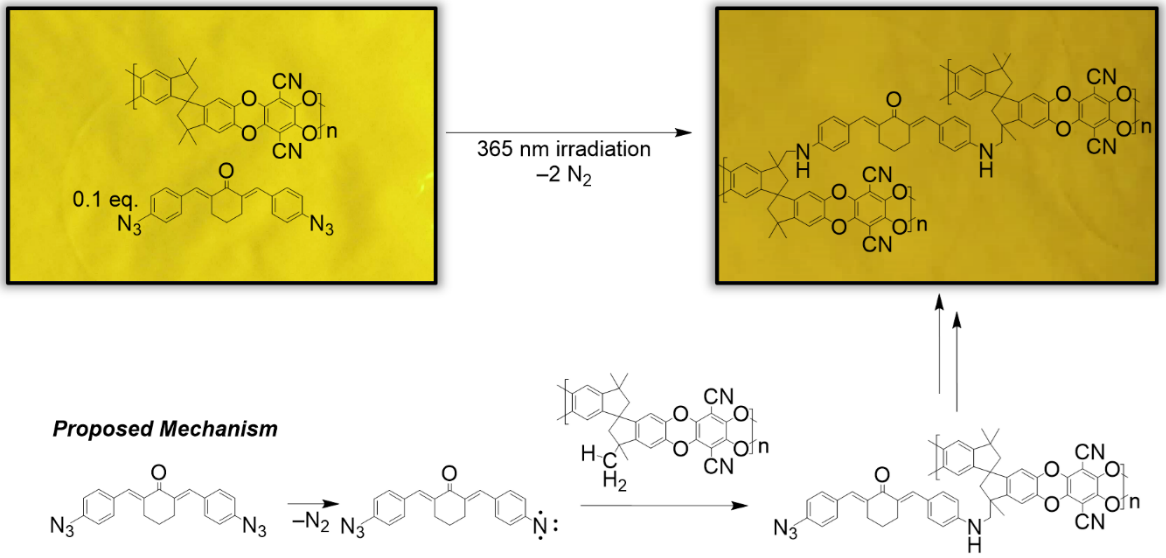

Figure S1. Schematic demonstrating the UV irradiation process invoked for the crosslinking of **PIM-1** coated separators with 2,6-bis(4-azidobenzylidene)cyclohexanone, and the proposed crosslinking mechanism.

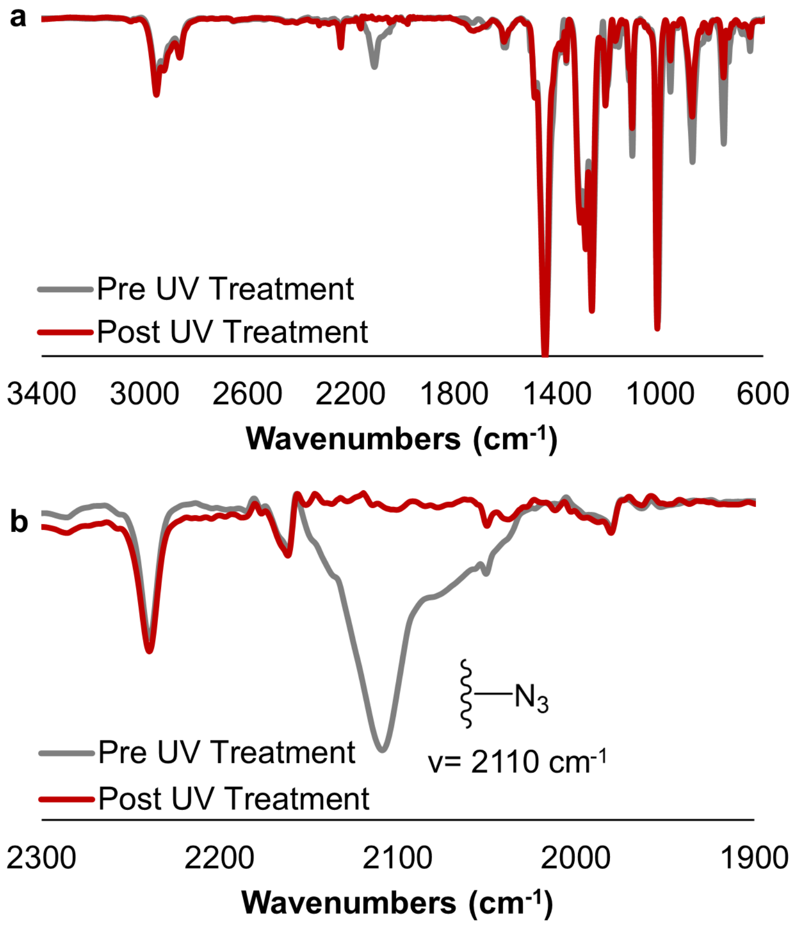

Figure S2. (a) FTIR spectrum of PIM-1-coated Celgard 2400 with 10 mol% 2,6-bis(4-azidobenzylidene)cyclohexanone before (grey) and after (red) exposure to 365 nm light for 30 min. (b) Expanded region of the spectrum to highlight consumption of the azide upon UV light exposure.

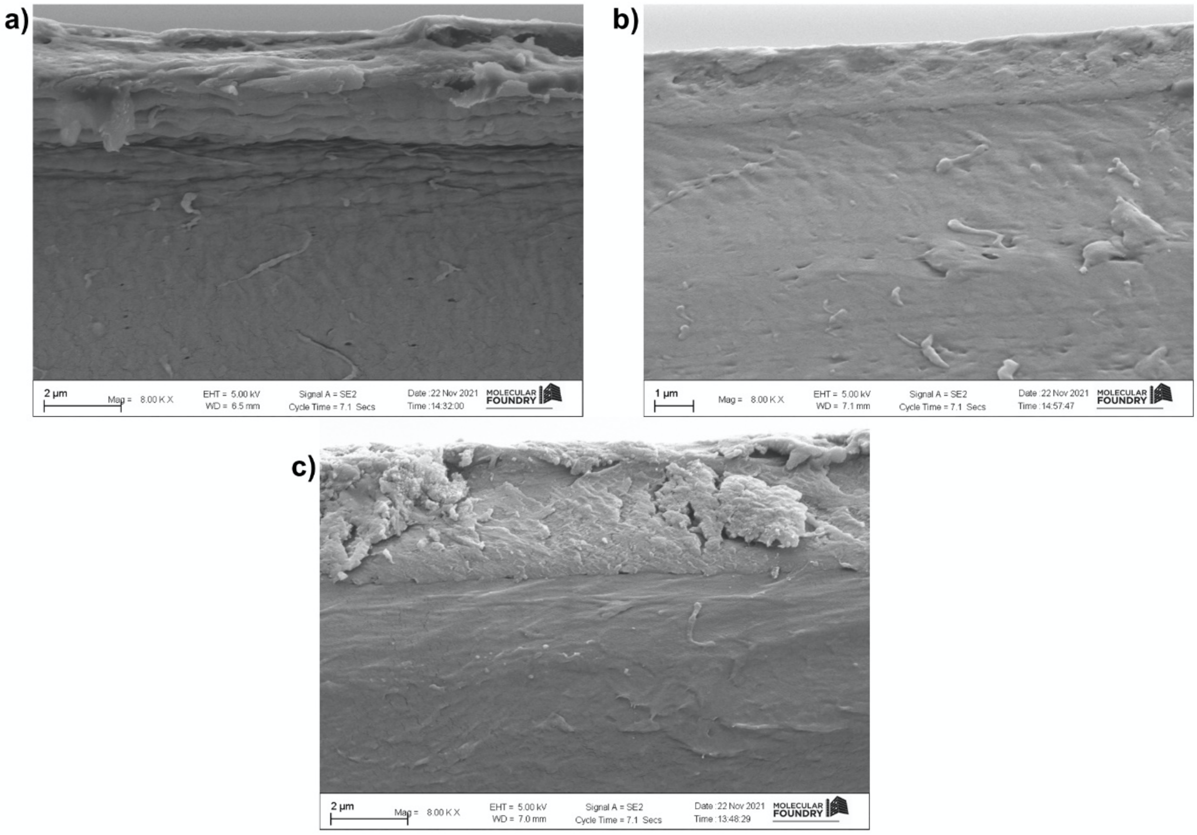

Figure S3. SEM images of polymer-coated separators used in crossover studies with a) **V(acac)$_3$**, b) **1-methyl**, and c) **1-pentyl**. Thicknesses are tabulated in Table S3.

Table S1. SEM measurements and Gurley numbers of **XPIM-1** coated separators used in this study.

Coated Separator[a]	Average Membrane Thickness ± SD (µm)	Celgard Thickness (µm)	Average Coating Thickness ± SD (µm)	Gurley Number (s)
a	28.332 ± 0.309	25	3.332 ± 0.309	10,200
b	27.056 ± 0.257	25	2.056 ± 0.257	8,360
c	28.241 ± 0.314	25	3.241 ± 0.314	9,840

[a] Coated separators are labelled as they appear in Figure S2.

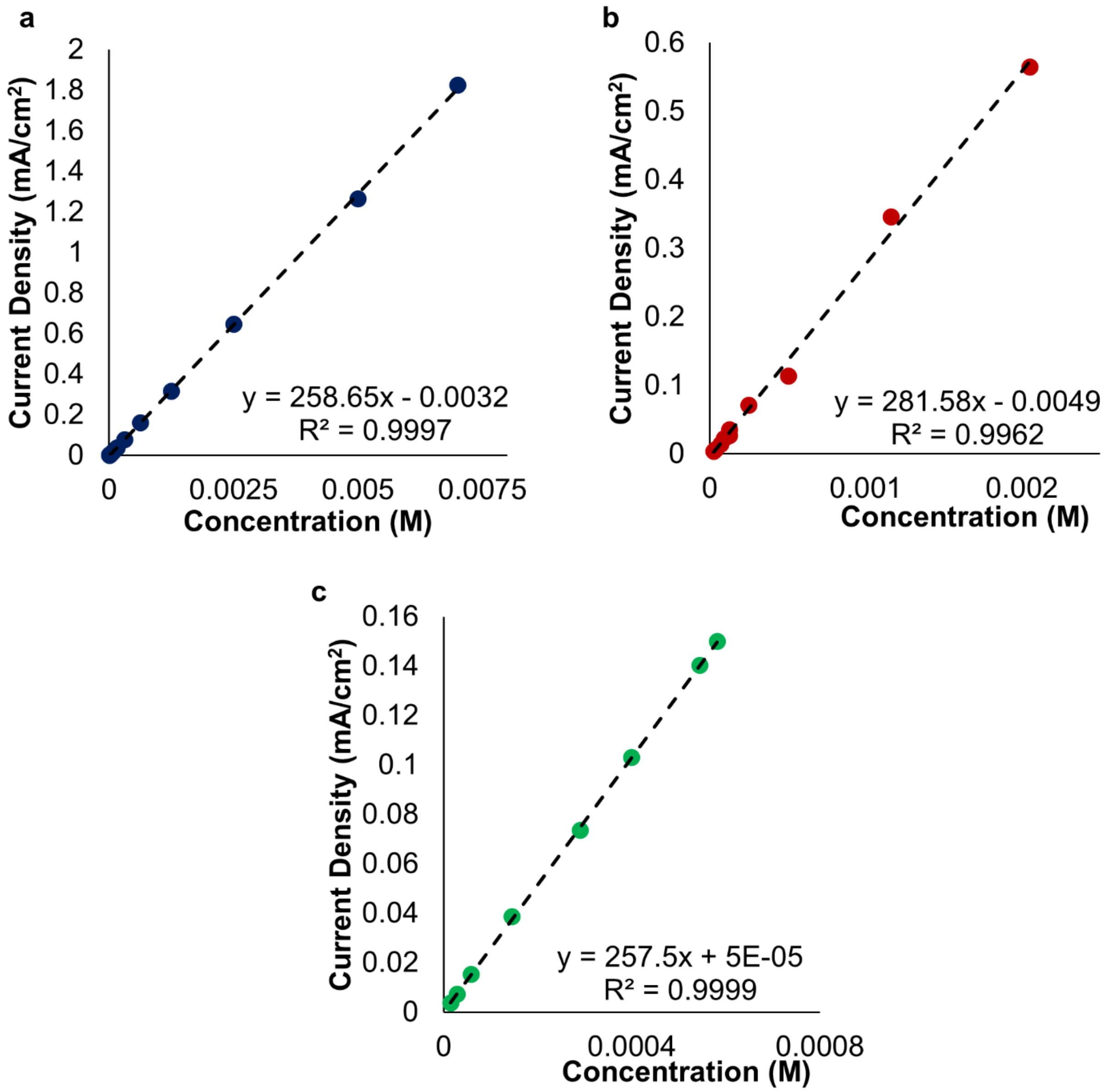

Figure S4. Calibration curves relating active species concentration and current density for the relevant redox events used in this study for a) **V(acac)$_3$**, b) **1-methyl**, and c) **1-pentyl**.

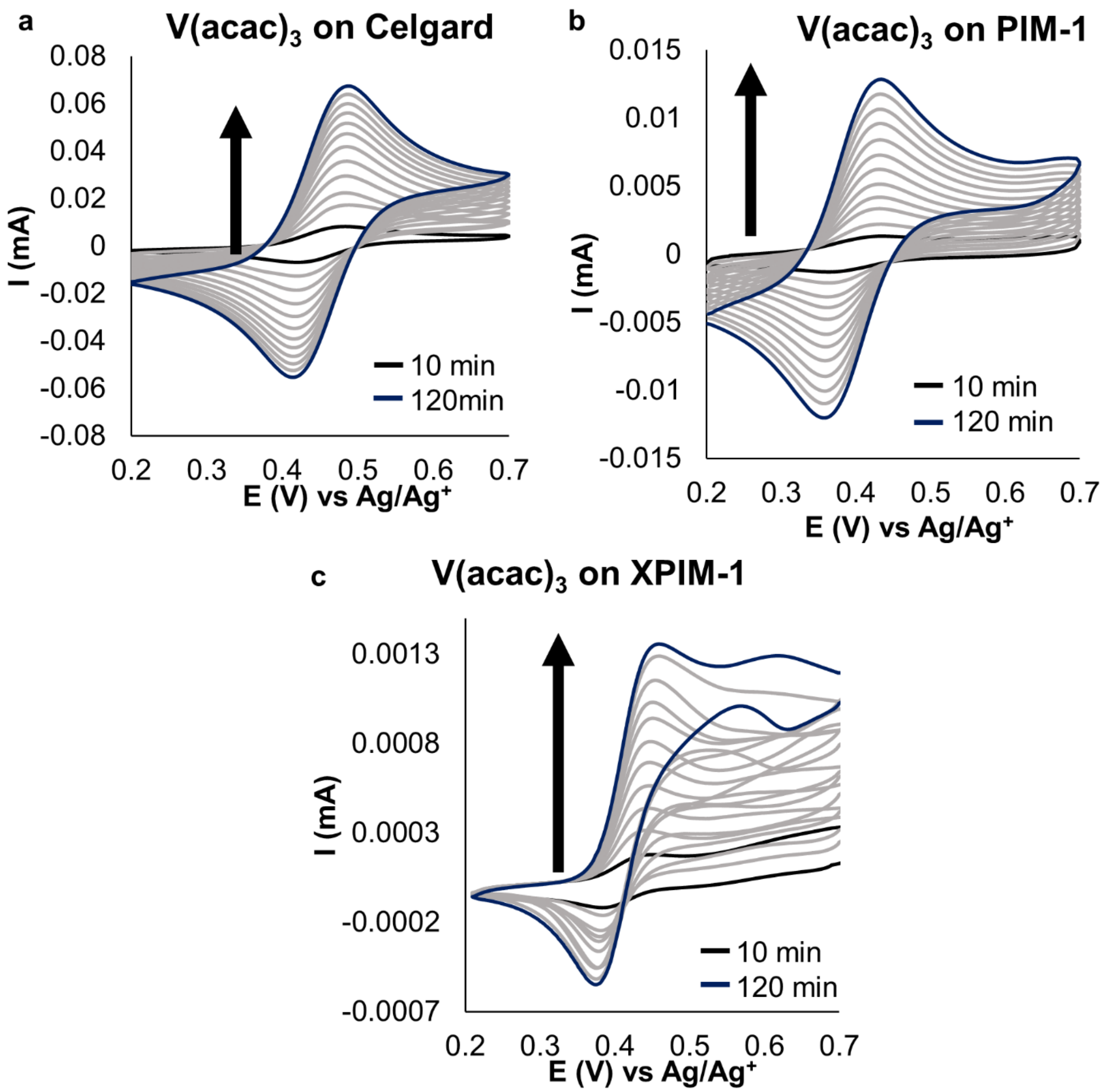

Figure S5. CV data collected in crossover experiments of **V(acac)$_3$** with a) bare Celgard 2400, b) **PIM-1**, and c) **XPIM-1** separators.

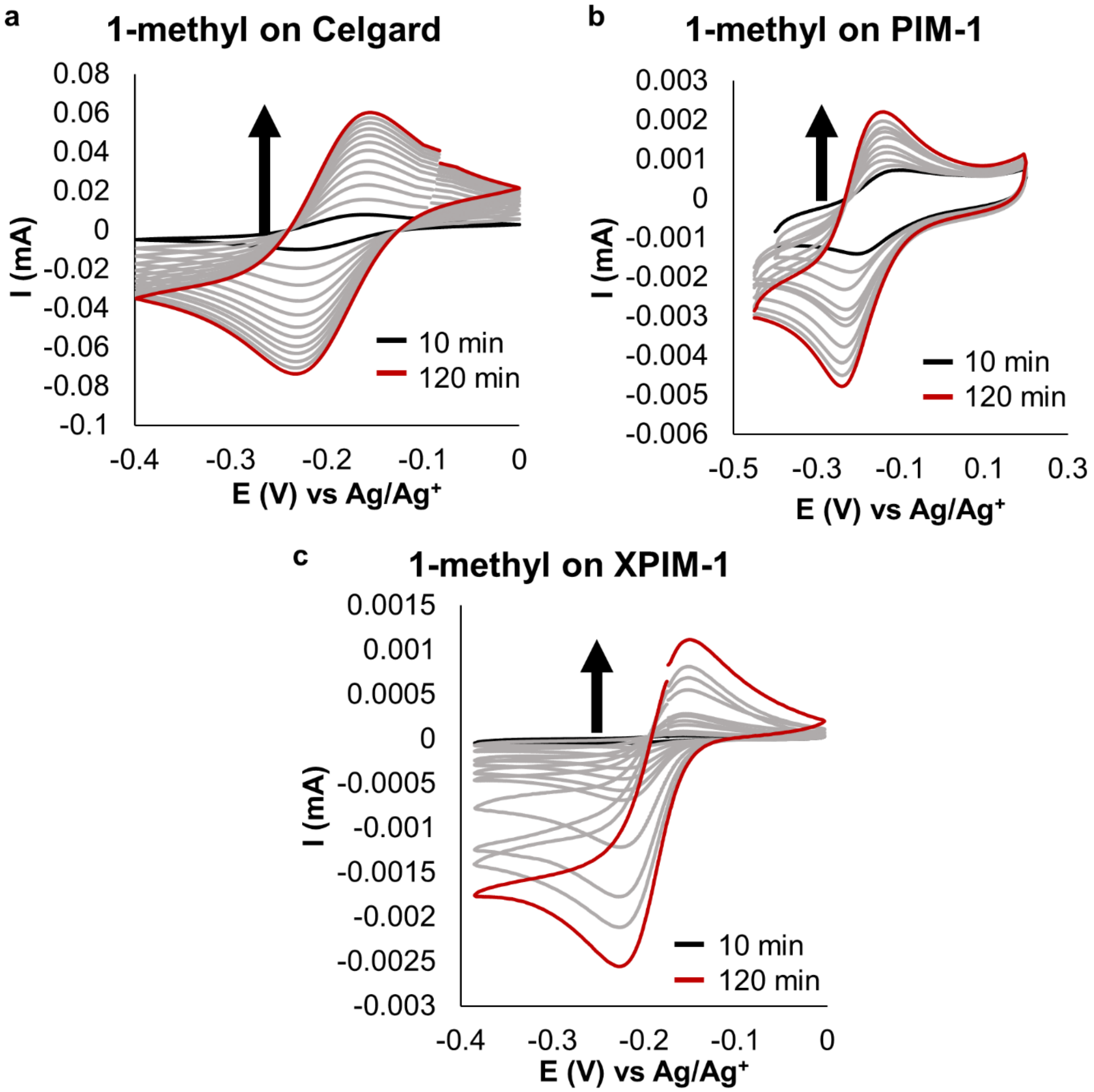

Figure S6. CV data collected in crossover experiments of **1-methyl** with a) bare Celgard 2400, b) **PIM-1**, and c) **XPIM-1** separators.

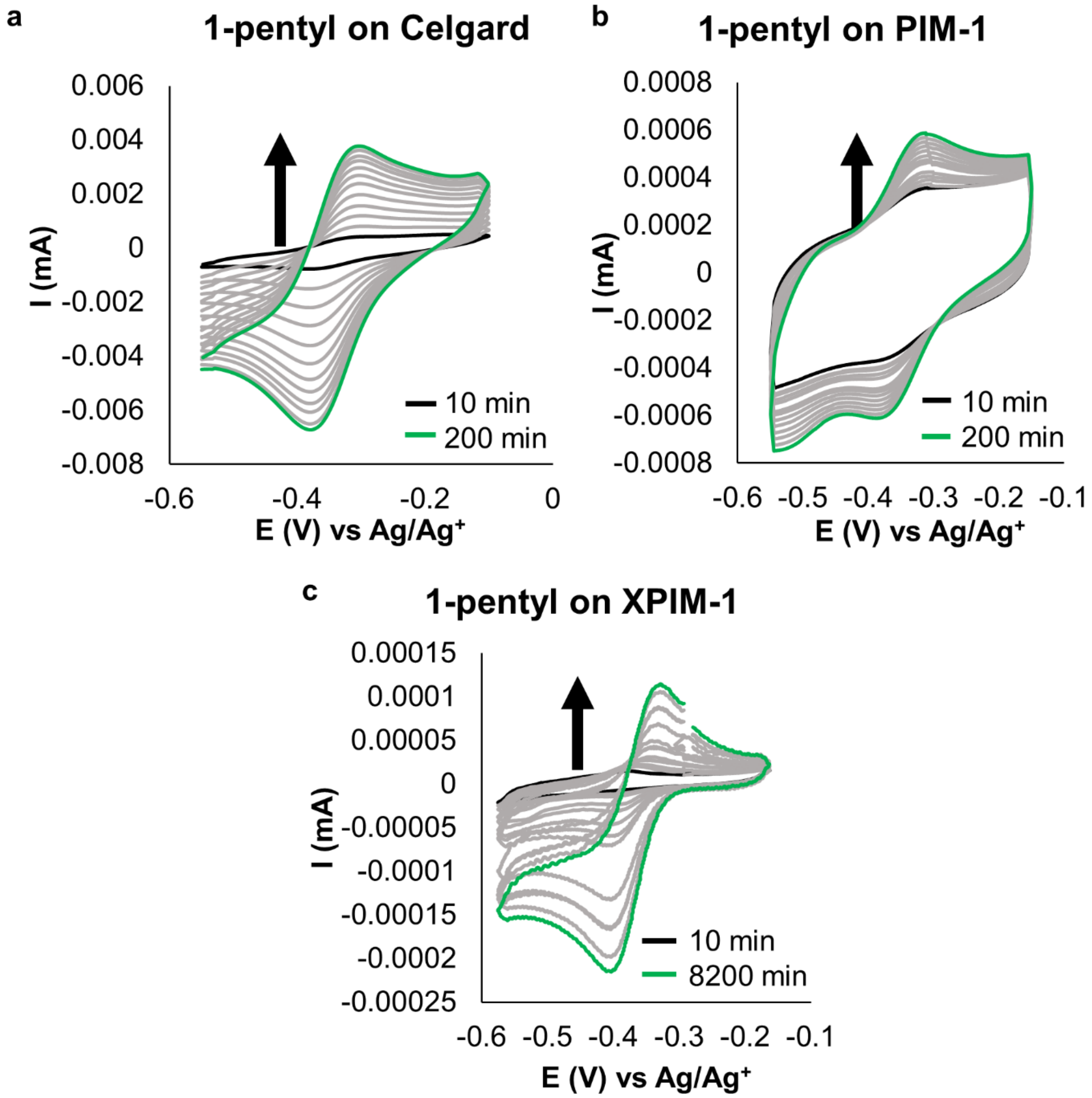

Figure S7. CV data collected in crossover experiments of **1-pentyl** with a) bare Celgard 2400, b) **PIM-1**, and c) **XPIM-1** separators.

Table S2. Crossover parameters for active species on Celgard 2400.

Compound	D_{sol} x $10^{6\,a}$ ($cm^2\,s^{-1}$)	Retentate Concentration (mM)	Membrane Thickness (cm)	D_{eff} x $10^{6\,b}$ ($cm^2\,s^{-1}$)	D_{sol}/D_{eff}
**V(acac)₃**	6.2[ref]	10	0.0025	0.975	6.36
**1-methyl**	1.4[ref]	10	0.0025	1.18	1.19
**1-pentyl**	1.76[ref]	4	0.0025	0.105	16.8

[a]D_{sol} is the diffusion coefficient of the inorganic complex through the MeCN electrolyte
[b]D_{eff} for Celgard is the membrane diffusivity of a compound through a bare Celgard 2400 sheet

Table S3. Crossover parameters for active species on **PIM-1**.

Compound	D_{sol} x $10^{6\,a}$ ($cm^2\,s^{-1}$)	Retentate Concentration (mM)	Membrane Thickness (cm)	D_{eff} x $10^{7\,b}$ ($cm^2\,s^{-1}$)	D_{eff} x $10^{8\,c}$ ($cm^2\,s^{-1}$)	D_{sol}/D_{eff}
**V(acac)₃**	6.2[ref]	10	0.00275	1.466	1.544	402
**1-methyl**	1.4[ref]	10	0.00275	0.5146	0.4871	287
**1-pentyl**	1.76[ref]	4	0.00275	0.05685	0.005437	3237

[a]D_{sol} is the diffusion coefficient of the inorganic complex through the MeCN electrolyte
[b]D_{eff} for Celgard is the membrane diffusivity of a compound through the composite PIM-coated separator
[c]D_{eff} for **PIM-1** and **XPIM-1** are the diffusivities of a complex through the polymer coating in isolation after accounting for diffusion through Celgard.

Table S4. Crossover parameters for active species on **XPIM-1**.

Compound	D_{sol} x $10^{6\,a}$ ($cm^2\,s^{-1}$)	Retentate Concentration (mM)	Membrane Thickness (cm)	D_{eff} x $10^{7\,b}$ ($cm^2\,s^{-1}$)	D_{eff} x $10^{8\,c}$ ($cm^2\,s^{-1}$)	D_{sol}/D_{eff}
**V(acac)₃**	6.2[ref]	10	0.0028332	1.083	1.408	440
**1-methyl**	1.4[ref]	10	0.0027056	0.4093	0.3214	436
**1-pentyl**	1.76[ref]	4	0.0028241	0.001685	0.01026	17150

[a]D_{sol} is the diffusion coefficient of the inorganic complex through the MeCN electrolyte
[b]D_{eff} for Celgard is the membrane diffusivity of a compound through the composite PIM-coated separator
[c]D_{eff} for **PIM-1** and **XPIM-1** are the diffusivities of a complex through the polymer coating in isolation after accounting for diffusion through Celgard.

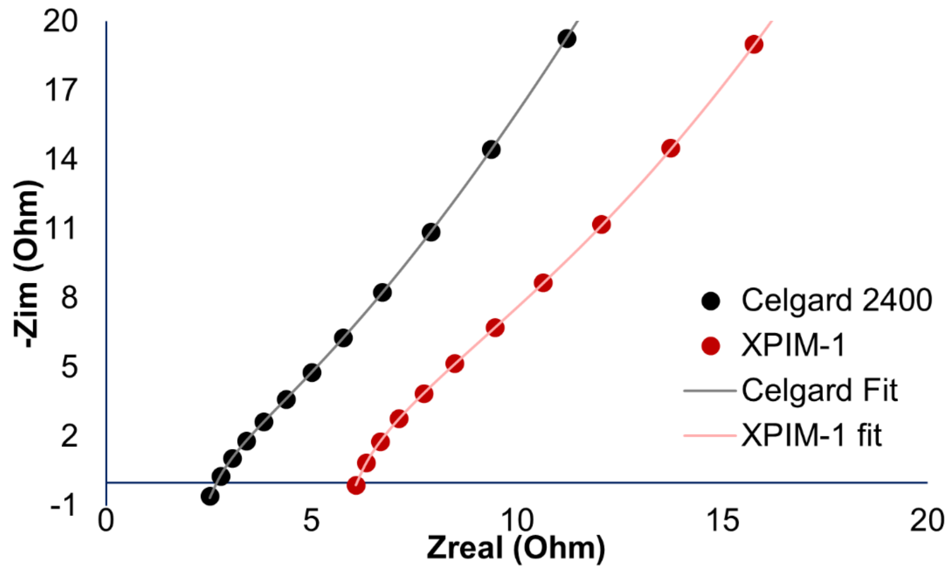

Figure S8. EIS Spectra of electrolyte-soaked Celgard 2400 (black circles) and **XPIM-1** (red circles) membranes.

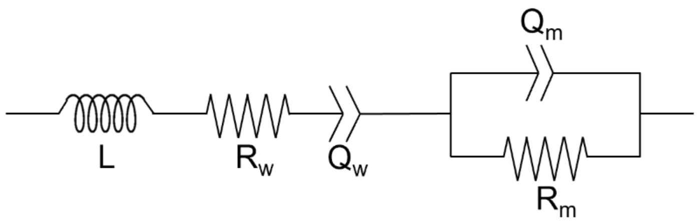

Figure S9. Equivalent circuit used for EIS fitting of spectra in Figure S8.

Table S5. EIS parameters for conductivity measurements on Celgard 2400 (black circles) and **XPIM-1** (red circles) membranes.

**Membrane**	**Resistance (R_m, Ohm)**	**Thickness (cm)**	**Electrode Area (cm^2)**	**Conductivity, σ (mS cm^{-1})**
**Celgard 2400**	2.699	0.0025	1.131	0.819
**XPIM-1**	6.119	0.00283	1.131	0.409

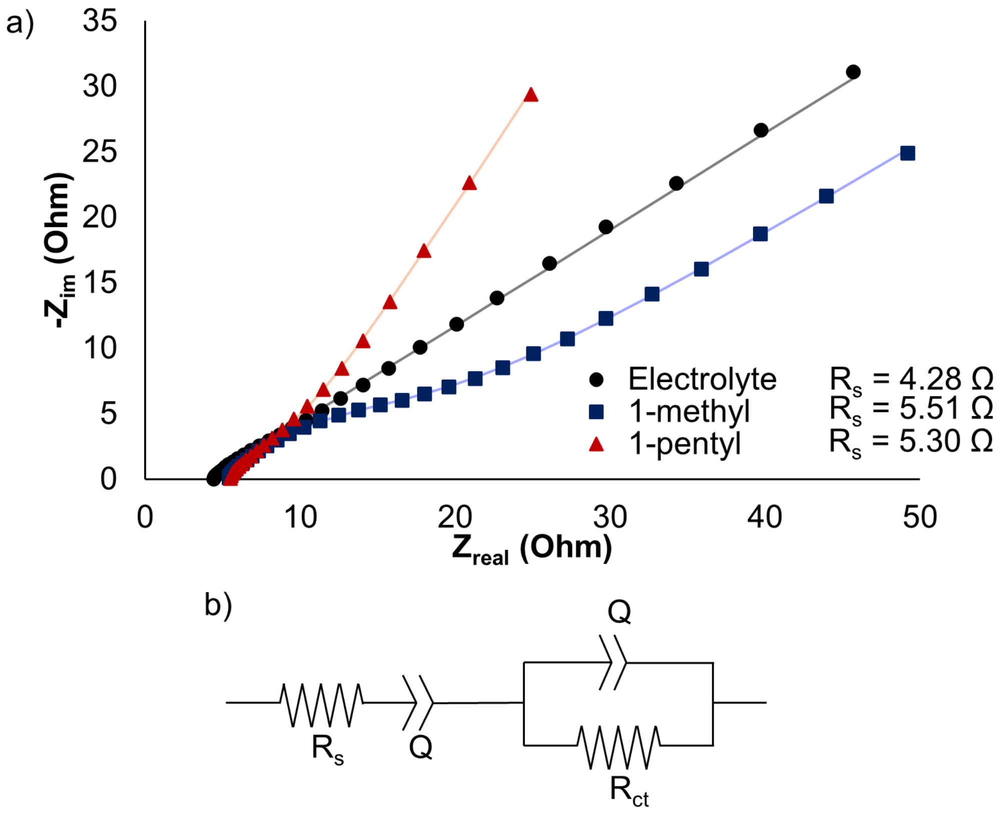

Figure S10. a) EIS spectra of electrolyte solutions of 0.1 M [^{n}Bu$_4$N]PF$_6$, as well as this electrolyte with 10 mM **1-methyl** and 4 mM **1-pentyl**, with their respective solution resistance (R_s) values. b) Equivalent circuit used to fit the EIS spectral data.
